# Salt Stress Alleviation in *Triticum aestivum* Through Primary and Secondary Metabolites Modulation by *Aspergillus terreus* BTK-1

**DOI:** 10.3389/fpls.2022.779623

**Published:** 2022-03-10

**Authors:** Muhammad Ikram Khan, Niaz Ali, Gul Jan, Muhammad Hamayun, Farzana Gul Jan, Amjad Iqbal, Anwar Hussain, In-Jung Lee

**Affiliations:** ^1^Department of Botany, Hazara University, Mansehra, Pakistan; ^2^Department of Botany, Abdul Wali Khan University, Mardan, Pakistan; ^3^Department of Food Science & Technology, Abdul Wali Khan University, Mardan, Pakistan; ^4^School of Applied Biosciences, Kyungpook National University, Daegu, South Korea

**Keywords:** endophytes, *Aspergillus terreus* BTK-1, phytohormones, salt stress, molecular identification

## Abstract

We report the growth promoting potential in wheat under saline conditions by an endophytic fungus *Aspergillus terreus* BTK-1. The isolated BTK-1 from the root of *Chenopodium album* was identified as *Aspergillus terreus* through 18S rDNA sequence analysis. BTK-1 secreted indole acetic acid (IAA), exhibited 1- aminocyclopropane-1- carboxylate deaminase (ACC) and siderophores activity, and solubilized phosphate. Wheat seedlings were exposed to a saline environment (0, 60, 120, and 180 mM) with or without BKT-1 inoculation. Seedlings inoculated with BTK-1 showed higher concentrations of IAA and gibberellins, whereas they showed low concentrations of abscisic acid compared to the BTK-1 non-inoculated plants. Also, BTK-1 inoculated wheat plants revealed significantly (*P* = 0.05) longer shoots and roots, biomass, and chlorophyll contents. On the contrary, plants without BTK-1 inoculation indicated significantly (*P* = 0.05) low amounts of carbohydrates, phenolics, prolines, potassium, magnesium, and calcium, with high amounts of Na and malonaldehyde under salt stress. Likewise, BTK-1 inoculated wheat plants showed high activity of reduced glutathione, and low activity of ascorbate, catalase, and peroxidase under salt stress. The mitigation of salinity stress by BTK-1 inoculated wheat plants suggested its use as a bio-stimulator in salt affected soils.

## Introduction

Environmental stresses are among the most limiting factors globally for agricultural productivity and threat for crop yield (Muhammad et al., 2018, 2019; Ismail et al., 2021). Saline soils are one of the major issues to achieve sustainable agriculture. The effect of salinity is more severe in arid and semiarid regions owing to low rainfall, temperature, water quality, high evapotranspiration, and poor soil management practices (Shrivastava and Kumar, 2015). Soil salinity is known to inhibit plant growth (Hamayun et al., 2015, 2017). In the current scenario, scientists around the planet are working on cheap and quick methods to enhance agricultural productivity and sustainability (Mbarki et al., 2018; Moghimi et al., 2018; Del Buono, 2021). In this regard, the use of beneficial microbial symbionts is an alternative way to improve agricultural productivity and sustainability under salt stress (Khushdil et al., 2019; Nusrat et al., 2019).

Plant–microbe interactions are thought to be a viable way to improve agricultural productivity and functioning of successful agricultural system. Plants contain a relatively diverse population of endophytes, which spend time in host tissues without producing disease (Bibi et al., 2018; Bilal et al., 2018; Ali et al., 2019). Such plant growth promoting (PGP) microbes are attaining great focus among plant biologists (Gul Jan et al., 2019; Jan et al., 2019; Kang et al., 2019; Qadir et al., 2020; Raid et al., 2021). Endophytic fungi, in fact, secured remarkable interest in the scientific community due to its symbiotic alliance with crop species (Ismail et al., 2018, 2019, 2020a,b,c, 2021). Endophytes help in plant fitness through fair distribution of growth-promoting hormones and nutrients, bring physical change in soil, and help in combating environmental stresses (Mehmood et al., 2019a,b). They produce an array of bioactive compounds that can be termed as plant growth-promoters (Kang et al., 2019). They have the ability to fetch nutrients, such as sulfur, potassium, calcium, phosphorus, and magnesium, from the soil to support host plant growth (Bilal et al., 2018; Kang et al., 2019). Endophytes produce siderophores and exhibit 1-aminocyclopropane- 1-carboxylate deaminase activity to scavenge ferric iron, fix nitrogen, and solubilize inorganic phosphate (Muhammad et al., 2018, 2019). Phytohormones produced by endophytes (for example auxins) can greatly influence metabolism, reproduction, and overall growth and help the host plant species exposed to various environmental stresses (Hamayun et al., 2017; Ismail et al., 2021; Raid et al., 2021). Certainly, it would not be an exaggeration to say that the endophytic association could recover plant health under biotic and abiotic stresses (Hamayun et al., 2017; Afridi et al., 2019; Muhammad et al., 2019; Ismail et al., 2021). Globally, wheat is considered as one of the most important cash crops. The non-uniform and rigorous use of chemical fertilizers to achieve high yields may lead to inconsistent supply of nutrients and low soil fertility. The raise in food demand at a steady rate requires a cheap and rigorous method to achieve high yields. Therefore, the current study was designed to evaluate the phytostimulatory characteristics and salt stress tolerance of a novel endophytic fungal isolate BTK-1 of *A. terreus*.

## Materials and Methods

### Study Area

Soil and plant samples were collected from the Kohat district that occupies the central part of Khyber Pakhtunkhwa (KPK). The Kohat district lies between 982429 m north latitude and 1057680 m east longitude.

### Soil Analysis

Soil samples were collected from the sampling sites (0–15 cm depth) with the help of a stainless-steel auger. The soil samples were dried in shade and then crushed, followed by sieving through 2 mm sieve. The resultant samples were stored in plastic bags till further physicochemical analysis. Textural classes were determined by the Bouyoucos method according to the USDA textural triangle (Bouyoucos, 1936). The electrical conductivity (EC) of the soil was determined at 25°C using an electrical conductivity meter (HM EC-3M). Soil pH was determined by using a Jenway pH meter (Model-3510). Lime contents of the soil were determined by EDTA titration method. The sodium concentration was extracted with 1 M ammonium acetate (NH_4_OAc) solution and estimated by a flame photometer (Jenway, PFP7/C) as described by Heald (1965).

### Plant Material and Isolation of Endophytic Fungi

*Chenopodium album* L. were collected from saline area of Kohat District Pakistan, and processed within 48 h of collection in the laboratory. Surface sterilization was carried out by the method of Muhammad et al. (2019). The root section of *C. album* was cut and washed in running tap water to remove soil and then soaked for 5 min in distilled water containing 4–5 drops of tween20. The soil-free soaked root samples were cut into pieces (0.5 cm) and surface sterilized by dipping it for 30 s in a solution containing 70% ethanol and 1% perchloric acid. The ethanol and perchloric traces were removed by rinsing the root segments with autoclaved distilled water (ddH_2_O). Sterilized root segments (5 segments per plate) were placed on Hagem medium (0.5% glucose, 0.05% KH_2_PO_4_, 0.05% MgSO_4_⋅7H_2_O, 0.05% NH_4_Cl, 0.1% FeCl_3_, 80 ppm streptomycin, and 1.5% agar; pH 5.6 ± 0.2) to isolate endophytic fungi. The plates were properly sealed and incubated at 27°C for 7 days. Intact surface sterilized roots were also processed in a similar way as root segments to check for any contamination by exogenous microbes. After 1 week of incubation, fungal hyphae were developed from the root segments. Individual colonies were collected and re-cultured on potato dextrose agar (PDA) media plates (potato infusion: 200 g, dextrose: 20 g, agar: 20 g, distilled water: 1 L). The PDA plates were incubated for a week at 25°C and this step was repeated till individual colonies were obtained. The fungal isolates were then enriched in flasks containing Czapek culture broth (1% peptone, 1% glucose, 0.05% MgSO_4_⋅7H_2_O, 0.05% KCl, 0.001% FeSO_4_⋅7H_2_O; pH 7.3 ± 0.2). The flasks were incubated in a shaking incubator for 7 days at 120 rpm and 30°C. The culture filtrate (CF) was harvested after centrifugation for 15 min at 4°C and 4,000 × *g*. The harvested pellets and supernatants were lyophilized (ISE Bondiro Freeze Dryer) at –70°C before further analysis.

### Screening of the Isolated Endophytes for Plant Growth Promotion

Indole acetic acid deficient maize mutant was initially used to screen the isolated fungal endophytes for phytostimulation and IAA production. The isolates were cultured in Czapek broth and incubated in shaking incubator for 7 days at 120 rpm and 27°C. Fungal mycelia (pellets) and culture filtrates (supernatant) were separated from each other after centrifugation (10,000 rpm) of the Czapek broth for 15 min at 4°C. The pellets (fungal biomass) were lyophilized and kept for the identification of potent fungal isolate(s), while the supernatants were used in maize growth promotion assays. IAA deficient maize seeds were surface sterilized as mentioned earlier and allowed to germinate in Petri plates containing moistened filter paper. The uniformly germinated seedlings were picked and grown in 0.8% water-agar medium under axenic conditions in growth chamber (photoperiod of 14 h light and 10 h dark; light intensity 1000 μmm-^2^s-^2^ Natrium lamps and relative humidity 60–70%). The fungal supernatant (10 mL) was sprayed at two-leaf stage on the apices of seedlings. Seedlings treated with distilled water were used as negative control. The seedlings from each treatment were harvested after 1-week of fungal supernatant application and evaluated for different growth parameters.

### Estimation of Indole Acetic Acid Produced by the Endophytic Fungal Isolate

High performance liquid chromatography (HPLC) chromatograph was used for the analysis of IAA in the supernatant of the potent fungal isolate. The fungal supernatant (20 μl) was loaded on a 5 μm reverse phase column (μBondapak C18, 250 mm × 4 mm) with the help of an HPLC micro – syringe. The sample was fractioned under isocratic conditions with methanol and water (80:20 v/v) as a mobile phase, at a flow rate of 1.0 ml/min. Eluates were detected by a differential ultraviolet detector at 254 nm. Pure IAA was used as a standard to quantify IAA in the fungal supernatant.

### Halotolerance Assay

The endophytic fungal isolate was enriched in the flasks containing Czapek broth media supplemented with various concentrations of salt (NaCl 50–500 mM). The flasks were transferred to the shaking incubator operated at 27°C and 120 rpm for 1 week. After incubation fungal mycelia from a broth media were filtered and fresh and dry weights were recorded.

### Plant Growth Promoting Assay

#### Phosphate Solubilization

Phosphate solubilization potential of the selected endophyte was detected on Pikovskaya’s (PVK) agar media by the method of Wahyudi et al. (2011). The PVK agar media was autoclaved and transferred to the plates. Fresh culture of the selected fungal stain was inoculated in the center of the plate under sterile conditions. The plates were incubated at 28 ± 2°C for 3 days; clear halo zones around the fungal colonies were recorded.

A disk (4 mm diameter) from fresh fungal culture was transferred to the Pikovskaya broth media in a 250 ml conical flask. The inoculated flasks were incubated at 30°C and 120 rpm for 15 days in a shaking incubator along with control (Un-inoculated flasks). After incubation, the contents of the flask was filtered (Whatman No. 42), and phosphate solubilization was estimated by the method used by Jackson (2005).

#### Siderophore Assay

The siderophore assay was performed on chrome azurol S agar media by following the modified protocol of Schwyn and Neilands (1987). The media was prepared by dissolving 60.5 mg of CAS in 50 ml distilled water, following the addition of 10 ml iron (III) solution (1 mM FeCl_3_.6H_2_O, 10 mM HCl) with persistent stirring. To this, 72.9 mg of hexadecyl tri methyl ammonium bromide (HDTMA) was added to the mixture and then topped with 40 ml of water. The contents were then autoclaved at 121°C for 15 min. After sterilization the media was added to the basal media [succinic acid 0.5%, K_2_HPO_4_ 0.4%, (NH_4_)_2_SO_4_.7H_2_O, agar 2% at pH 5.3 ± 2], and gently mixed till the appearance was of a blue color. After the development of a blue color the media was transferred to the Petri plates. The fresh fungal culture was inoculated on Chrome azurol S agar media and incubated at 27°C for a week. The zone diameter was determined by the change in color from blue to purple, dark purplish red, or yellowish orange around the fungal colonies.

### Analysis of the 1-Aminocyclopropane-1-Carboxylate Deaminase Activity

1-Aminocyclopropane-1-carboxylate deaminase (ACC) enzyme assay was performed by using the protocol of Penrose and Glick (2003). Liquid Dworkin and Foster mineral medium (Dworkin and Foster, 1958) was prepared (3.0 mM ACC), and then the fresh fungal culture was added to the medium and incubated at 30°C and 200 rpm for 48 h to determine the ACC deaminase production. The supernatant from the culture medium was collected at 12, 24, 36, and 48 h. The amount of α- ketobutyrate in medium was measured at 540 nm absorbance along with standard α- ketobutyrate (Sigma-Aldrich Co., Rockville, MD, United States) ranging between 0.1 and 1.0 μM. The ACC deaminase activity was expressed as the amount of α- ketobutyrate produced per mg of protein per hour.

### DNA Extraction and Fungal Isolate Identification

Genomic DNA isolation and PCR were performed according to an established protocol of Khan et al. (2008). Selected endophytic fungal isolate was identified by amplifying their ITS region of 18 S rDNA with universal primers, NS1 5′ (GTA GTC ATA TGC TTG TCT C) 3′ and NS24 5′ (AAA CCT TGT TAC GAC TTT TA) 3′. The BLAST search program^1^ was used to compare the sequence homology of the nucleotide of the 18S region of fungi. The closely related sequences obtained were aligned through CLUSTAL W using MEGA version 4 software (Tamura et al., 2004), and the maximum parsimony tree was constructed using the same software. The bootstrap replications (1,000) were used as a statistical support for the nodes in the phylogenetic tree.

### Wheat-Endophytes Interaction Under Salt Stress

Seeds of wheat variety Bhakkar-2000 (KJ672075) were obtained from the National Agricultural Research Center (NARC), Islamabad. Healthy seeds were sterilized (70% ethanol and 1% perchloric acid), germinated in petri plates, and incubated at 28°C for 24 h. To study the microbe’s interaction in wheat plants under salt stress, uniformly germinated seedlings were transferred from petri plates to autoclaved pots containing 3 kg soil. The examined composition of the soil was sand (54%), silt (44%), and clay (1.6%) with water holding capacity of 220 ml water/Kg soil (± 3.5), pH (6.8), soil texture (loamy sand), lime (2.5%), and soil organic matter (1.6%). The experiment was performed in triplicate; each replicate contained 10 pots and each pot consisted of six seedlings (total = 6 × 10 × 3 = 180 seedlings per treatment). The seeds treated with non-inoculated media were used as a control. The pots were kept in a greenhouse (30°C, 80% humidity, light/dark 14 h/10 h). To prepare inoculum of the selected fungal isolate, mycelium was harvested by centrifugation at 5000 × *g* and 4°C for 15 min from 7-day-old fungal culture. To each pot, 50 mg of crushed fungal mycelium was added and the pots were kept in a controlled environment to acclimatize the fungal isolate. The treatments were divided in to four:

T1 = Control = 0 mM NaCl (No NaCl).
T2 = 60 mM NaCl (low NaCl concentration).
T3 = 120 mM NaCl (Moderate NaCl concentration).
T4 = 180 mM NaCl (High NaCl concentration).

Salt treatments were applied after 3 weeks of germination. Seedlings from salt treatments were watered with 100 ml salt solution at 12 h intervals for the next 7 days and seedlings from the control treatment were watered with distilled water.

### Analysis of Plant Growth Attributes

Randomly, 10 plants from each treatment were selected and leaves and roots were detached and kept separately. Root segments were carefully washed, scanned, and analyzed with WinRHIZO software (Ver 5.0, Regent Instruments Inc., Qubec, Canada) to determine root length, diameter, root surface area, and average root volume. The stomatal conductance and net photosynthetic rate were measured with a photosynthesis system (Li-6400, Li-COR Inc., Lincolin, NE, United States) in three plants per treatment. The stomatal conductance and net photosynthetic rate were measured under 1500 mmol/ms^2^ light intensity, 65% relative humidity, 32–62°C leaf temperature, and CO_2_ concentrations at 9:30–11:00 AM. Leaf area of mature leaves was measured with laser meter (CI-203 model, United States). The selected shoot and roots were washed with deionized water and dried at 70°C for 48 h to determine dry weight.

### Biochemical Attributes

#### Estimation of Chlorophyll and Carotenoid Content

To determine chlorophyll and carotenoids contents, segments (0.8-cm diameter) from fully expanded leaves were cut out in the form of disks. The leaf disks were mixed with 2 mL of acetone (80%) and washed twice with a further 2 mL of acetone. The absorbance of the extracts was measured using a spectrophotometer (UV-1700 Pharma Spec; UV-VIS; Shimadzu Japan) at 480, 645, and 663 nm. The contents of Chl *a*, Chl *b*, and carotenoids in the extracts were determined by using MacKinney equations (Mackinney, 1941).

#### Proline Estimation

Proline content was observed according to the method of Bates et al. (1973). The collected fresh healthy mature leaves were washed properly with distilled water. The clean samples (200 mg) were crushed in 3% sulfo salicylic acid and centrifuged for 5 min at 13,000 rpm. The supernatant (2 ml) was collected and mixed with glacial acetic acid (2 ml) + acid ninhydrin (2 ml). The mixture was boiled in a water bath at 100°C for 1 h and then immersed immediately in ice to stop the reaction. Toluene (1 ml) was added to the reaction mixture and the mixture was shaken for 20–30 s. The absorbance of the mixture was measured by spectrophotometer at 520 nm.

#### Estimation of Total Phenolics

Phenolic content was estimated by the method of Malik and Singh (1980). Fresh leaves of wheat plants were collected and air dried in shade at ambient temperature. The dried leaves (200 mg) were ground into powder and mixed with methanol (1 ml) in eppendorf tube. The contents of the tube were kept for 4 h before 6 min of centrifugation at 5000 rpm. The supernatant (methanol extract 0.5 ml) was collected in fresh tubes and mixed with 2.5 ml of 10% FC reagent. The resultant mixture was retained for 5 min before mixing with 2.5 ml distilled water + 2.5 ml sodium hydrogen carbonate (7.5% NaHCO_3_). After complete mixing the contents were kept for 1 h in the dark. The absorbance was measured at 650 nm after appearance of a blue color. The 10% FC reagent was used as blank. The total phenolic contents in the sample were measured from the calibration plot and expressed as mg galic acid equivalent of phenol/g of sample.

### Total Carbohydrate Contents Estimation

The carbohydrate contents in wheat plants were observed following the protocols of Hedge and Hofreiter (1962). Healthy mature leaves from treatment plants were collected and dried. The dried plant material (200 mg) was taken in test tube, to which 10 ml water was added and boiled in a water bath for 1 h. The extract (1 ml) from the test tubes was mixed with 3 mL of 3% antheron reagent and kept on a water bath operated at 100°C for 30 min. The final absorbance was measured at 630 nm.

### Malonaldehyde Determination

Malonaldehyde (MDA) was determined by following the method of Hodges et al. (1999). Fresh leaf samples (0.2 g) were homogenized in 10 ml of 10% trichloroacetic acid. The homogenate was centrifuged at 12000 rpm for 10 min and the supernatant was carefully pipetted in to a new tube. Thiobarbituric acid (2 ml of 0.6%) was added to the supernatant (2 ml) and the mixture was incubated in a water bath for 15 min at 100°C. The mixture was cooled and centrifuged at 12,000 rpm for 10 min. The absorbance of the supernatant was calculated at 532, 600, and 450 nm.

### Estimation of Enzymatic Activities

The fresh healthy leaves of experimental wheat plants were collected and homogenized in extraction buffer (5 mL) containing 50 mm sodium phosphate (pH 7.8). The sample was centrifuged and the supernatant was applied to measure the activities of catalase following the methods of Aebi (1984). GSH activity was determined by the method of Carlberg and Mannervik (1975). The activity of ascorbate peroxidase was measured according to the method of Nakano and Asada (1981).

### Determination of Phytohormones

Plant hormones [gibberellins (GAs), abscisic acid (ABA), and IAA] extraction and purification in different treatments were quantified on HPLC by the method of Kettner and Dörffling (1995). The samples were passed through Millipore filter (0.45 μ) and were analyzed on HPLC (Agilent 1100) equipped with variable UV detector and C18 column (39 × 300mm) (BondaPack Porasil C18, 37/50 μm, Waters, Eschborn, BRD). Methanol and water in the ratio of (30:70; v/v) were used as mobile phase @ 1500 μl/min with a run time of 20 min/sample. The plant hormones were identified on the basis of retention time. IAA and GA were eluted at 280 and 254 nm wavelengths, respectively. For ABA the samples were injected onto a C18 column and eluted with a linear gradient of methanol (30–70%), containing 0.01% acetic acid, at a flow rate of 0.8 ml min^–1^.

### Minerals Analysis

The concentration of ions (Ca, Mg, K, Na) were quantified in dried ground plant samples. Approximately, 0.5 g of ground dried plant materials were put in digesting tubes, to which was added 3 mL perchlorate acid and 10 mL of concentrated nitric acid. The samples were then soaked for 12 h and burned at 300°C for 3 h. The residue was transferred to a 50-mL volumetric flask and had 50 mL distilled water added. The ion contents was then measured using an atomic absorption spectrophotometer (TAS-986; PERSEE Ltd., Beijing, China) (Ashraf and Orooj, 2006).

### Statistical Analysis

Different treatments were compared by one way ANOVA at *P* = 0.05, followed by Tukey’s multiple comparison tests by Graph Prism 5 software. The BLAST search program (see footnote 1) was used to compare the sequence homology of the nucleotide of the 18S ITS region and were aligned through CLUSTAL W using MEGA version 4 software.

## Results

### Physiochemical Properties of the Soil

Soils were collected from the Kohat district of Khyber Pakhtunkhwa (KPK) Pakistan, from two depths (0–20 and 20–40 cm) for physico chemical analysis. Sand content of the surface soil (0–20 cm) ranged 2.01–76%, while at subsurface (20–40 cm) it was 12.4–80%. Silt content at depth 0–20 cm ranged from 10.7 to 70%, while at depth of 20–40 cm, it was 5–55%. Clay content at soil surface was 11–58.6%, while at subsurface clay contents varied from 1.9 to 69.8% (Table 1). Soil pH ranged from 7.1 to 7.9 in a soil depth from 0 to 20 cm, while at subsoil, the pH varied from 7.1 to 8.0. Electrical conductivity of the soil surface ranged from 2.8 to 18.2%, while at sub surface it varied from 4.4 to 17.1%. Lime content of the surface soil ranged from 1.87 to 30.1%, while at subsurface it was 2.7–38.09%. Lime contents ranged from slightly calcareous to strongly calcareous in both depths (Table 1).

**TABLE 1:** Physio-chemical properties of Kohat district KPK Pakistan.

Soil texture	Depth [cm]	Range	%	Soil nature	Depth [cm]	Range	%
Sand	0–20	Min	2.01%	Soil pH	0–20	Min	7.1
		Max	76.0%			Max	7.9
	20–40	Min	12.4%		20–40	Min	7.1
		Max	80.0%			Max	8.0
Silt	0–20	Min	8.45%	Electrical conductivity (μs/cm)	0–20	Min	2.8
		Max	83.0%			Max	18.2
	20–40	Min	6.00%		20–40	Min	34.7
		Max	60.0%			Max	31.7
Clay	0–20	Min	1.00%	Lime content (%)	0–20	Min	11.9
		Max	8.60%			Max	30.1
	20–40	Min	9.00%		20–40	Min	38.1
		Max	9.80%			Max	72.7

### Screening Bioassay for Fungal Isolates

A total of 10 fungal endophytes was isolated from the roots of *C. album* plants. The fungal isolates were identified on the basis of macroscopic characteristics (such as shape, colony height, growth rate and microscopic characteristics aerial hyphae, surface texture, base color, and margin). Morphological analysis showed that out of total isolated endophytes only five isolates were different. Culture filtrates (CF) of identified fungal isolates were tested on IAA mutant (dwarf cultivar) maize seedlings to identify the potent growth stimulatory and phytohormones producing fungi. The growth traits of mutant maize seedlings were recorded after 1 week of culture inoculation. Among the endophytic fungal isolates, BTK-1 enhanced shoot and root growth, chlorophyll content, and fresh biomas of IAA dwarf maize seedlings compared to the control seedlings. Other tested isolates showed less improvement in shoot growth, fresh weight, and chlorophyll content of mutant maize seedlings. Based on significant (*P* = 0.05) growth promoting potential, the endophytic fungal isolate BTK-1 was selected for identification and further analysis.

### Salt Resistance by BTK-1

**FIGURE 1:**
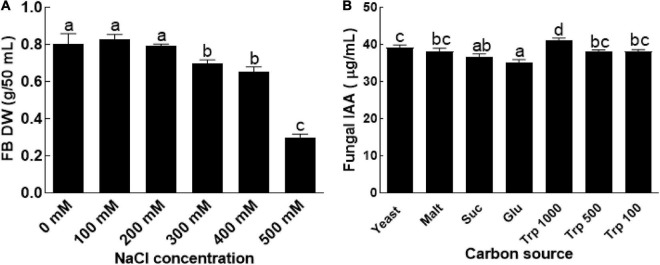
Salt tolerance ability and IAA production by endophytic fungal isolate BTK-1. **(A)** Represents salt resistance assay of endophytic fungal isolate BTK-1 cultivated under various concentrations of NaCl; **(B)** represents production of IAA by CGF-11 on media supplemented with different nutrient sources; Suc, sucrose; Glu, glucose; Trp, tryptophan; FB, fungal biomass; DW, dry weight. Means followed by different letters are significantly different at *P* ≤ 0.05. Each bar shows ± SE of means.

The results revealed that BTK-1 has resisted the salt stress up to 300 mM NaCL (Figure 1A). However, further increase in salt concentration resulted in growth reduction of endophytic fungal isolate BTK-1 as compared to the control. Increase in salt concentration negatively affected the fresh weight and dry weight of the BTK-1. At 500 mM NaCl in culture media, a clear decline was observed in both fresh (65%) and dry weights (60%) of endophytic fungal isolate BTK-1 compared to the control media. Minimal inhibitory concentration of NaCl was around 300 mM that reduced approximately 10% of the fresh and dry weight of endophytic fungal isolate BTK-1 (Figure 1A).

### Determination of IAA in the Culture Filtrates of BTK-1

Different concentrations of IAA (35–40 μg/ml) in different media with or without tryptophan were observed (Figure 1B). IAA production was most abundant in culture filtrates with different concentrations of tryptophan. The quantity of IAA in the culture filtrates of endophytic fungal isolate BTK-1 was highest (40 μg/ml), when the media was supplemented with 1000 μg tryptophan (Figure 1B).

### Estimation of Phosphate Solubilization, Siderophore, and 1-Aminocyclopropane-1-Carboxylate Deaminase

**FIGURE 2:**
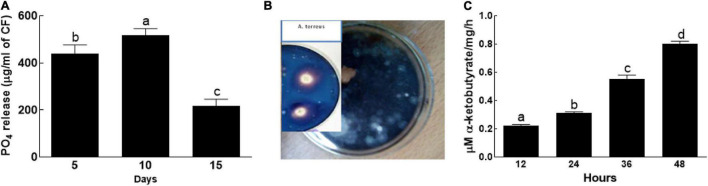
Phosphate solubalization, siderophore, and ACC activities of endophytic fungal isolate BTK-1. **(A)** Phosphate solubalization; **(B)** siderophore; **(C)** 1-aminocyclopropane-1-carboxylate (ACC) activity of BTK-1. Means followed by different letters are significantly different at *P* ≤ 0.05. Each bar shows ± SE of means.

The endophytic fungal isolate BTK-1 actively solubilized phosphate on exposure to PVK agar medium (Figure 2). The phosphate content of the BTK-1 CF were initially increased and then decreased during the incubation period (5–15 days). The amount of P content on 5th day of incubation was 440 μg/ml of CF, which increased to 517 μg/ml of CF on 10th day and then decreased to 217 μg/ml of CF on 15th day of incubation (Figure 2A). Furthermore, the endophytic fungal isolate BTK-1 also exhibited siderophore activity (Figure 2B). Clear yellow halozones around the BTK-1 colonies were observed. Also, ACC deaminase activity was recorded for the endophytic fungal isolate BTK-1 that ranged from 0.7 to 0.9 μM α-ketobutyrate/mg/h during the incubation time (Figure 2C).

### Identification of Endophytic Fungal Isolate BTK-1

**FIGURE 3:**
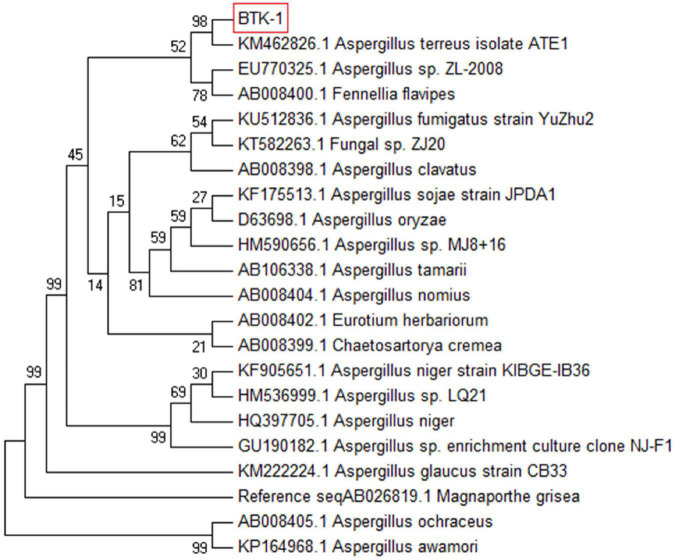
Multiple sequence alignment file of 18S ribosomal DNA region.

The lyophilized culture of the endophytic fungal isolate BTK-1 was amplified by PCR and identified through phylogenetic analysis of the 18S rDNA sequence. Based upon BLAST results, a sequence of 18S rDNA of endophytic fungal isolate BTK-1 showed maximum homology (98%) with *Aspergillus terreus*. To confirm the identity of the endophytic fungal isolate, phylogenetic consensus tree was constructed by using maximum parsimony (MP) method in MEGA 7 package (Figure 3). The sequence of BTK-1 was submitted to GenBank under accession No MF678562.

### Effect of BTK-1 on Wheat Growth Under Salt Stress

**FIGURE 4:**
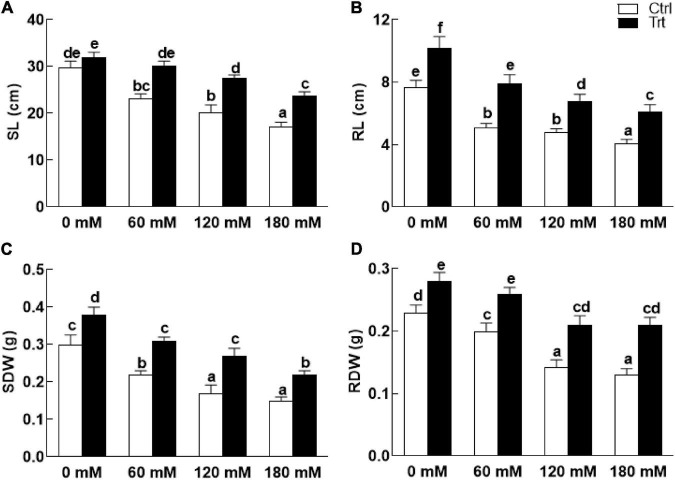
Effect of endophytic fungal isolate BTK-1 on growth parameters of wheat plants under salt stress. Salt stress was given after 3 weeks of seed germination. **(A)** Represents shoot length (SL); **(B)** represents root length (RL); **(C)** represent shoots dry weight (SDW); **(D)** represent roots dry weight (RDW); Ctrl, control plants; Trt, BTK-1 inoculated plants; Means followed by different letters are significantly different at *P* ≤ 0.05. Each bar shows ± SE of means.

**FIGURE 5:**
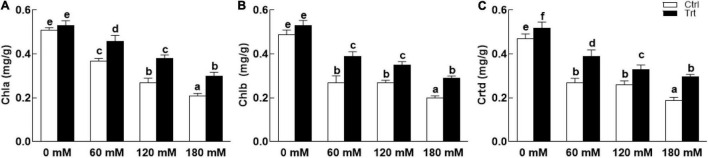
Effect of endophytic fungal isolate BTK-1 on chlorophylls and carotenoids contents of wheat plants under salt stress. Salt stress was given after 3 weeks of seed germination. **(A)** Represents chlorophyll *a* (Chla) contents; **(B)** represents chlorophyll *b* (Chlb) contents; **(C)** represents carotenoid contents; Ctrl, control plants; Trt, BTK-1 inoculated plants; Means followed by different letters are significantly different at *P* ≤ 0.05. Each bar shows ± SE of means.

Wheat seedlings grown in pots inoculated with BTK-1 mycelium showed a significant (*P* = 0.05) improvement in shoot and root lengths and weights compared to the non-inoculated wheat plants under normal as well as salt stress (Figure 4). Shoot lengths of BKT-1 associated wheat plants under salt stress (60, 120, 180 mM NaCl) were significantly (*P* = 0.05) improved compared to the BKT-1 non-associated wheat plants (Figure 4A). Similarly, root length (Figure 4B), shoot dry weight (Figure 4C), and root dry weight (Figure 4D) of BKT-1 associated wheat plants exposed to salt stress (60, 120, 180 mM NaCl) improved significantly (*P* = 0.05) compared to the BKT-1 non-associated wheat plants. The amounts of photosynthetic pigments (chlorophyll *a,b*/carotenoids) improved significantly (*P* = 0.05) in fungus-inoculated wheat plants as compared to the non-associated plants under normal as well as saline conditions (Figure 5).

### Effect of BTK-1 on Wheat Biochemical Attributes Under Salt Stress

**FIGURE 6:**
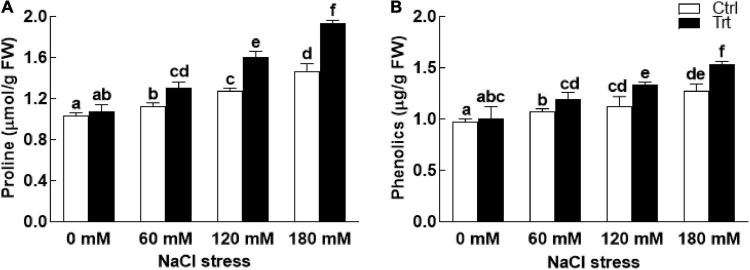
Effect of endophytic fungal isolate BTK-1 on proline and phenolic contents of wheat plants under salinity stress. Salt stress was given after 3 weeks of seed germination. **(A)** Represents proline contents; **(B)** represents phenolic contents; Ctrl, control plants; Trt, BTK-1 inoculated plants; Means followed by different letters are significantly different at *P* ≤ 0.05. Each bar shows ± SE of means.

**FIGURE 7:**
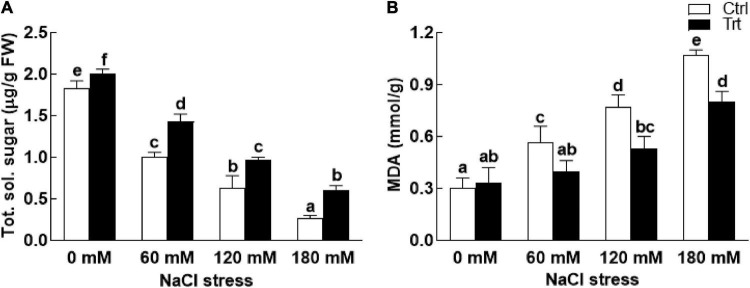
Effect of endophytic fungal isolate BTK-1 on total sugars and MDA contents of wheat plants under salt stress. Salt stress was given after 3 weeks of seed germination. **(A)** Represents total soluble sugars (Tot. sol. Sugar); **(B)** represents malondialdehyde (MDA) content; Ctrl, control plants; Trt, BTK-1 inoculated plants; means followed by different letters are significantly different at *P* ≤ 0.05. Each bar shows ± SE of means.

Under salt stress conditions (60, 120, 180 mM NaCl), proline and phenolic contents of the BTK-1 associated wheat plants were significantly (*P* = 0.05) high compared to the non-associated plants (Figure 6). The biosynthesis of proline and phenolic contents were gradually increased with an increase in salt concentration. Similarly, salt stress (60, 120, 180 mM NaCl) also inhibited the production of total carbohydrate contents in BTK-1 non-inoculated wheat plants compared to the BTK-1 inoculated plants (Figure 7A). Moreover, total carbohydrate contents were same in BTK-1 inoculated and non-inoculated wheat plants under normal condition (0 mM NaCl stress). On a general basis, treating wheat plants with different concentrations of NaCl (60, 120, and 180 mM) hindered the production of total carbohydrate contents in wheat plants (Figure 7A). The data regarding MDA content revealed a significantly (*P* = 0.05) low amount in BKT-1 associated wheat plants under salinity (60, 120, 180 mM) stress compared to the non-associated plants (Figure 7B).

### Effect of BTK-1 on Wheat Enzymatic Activities Under Salt Stress

**FIGURE 8:**
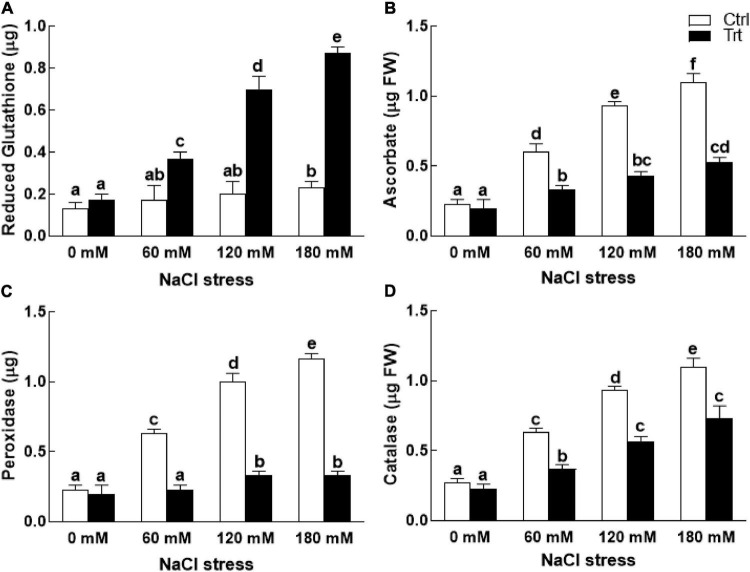
Effect of endophytic fungal isolate BTK-1 on the enzymatic activity of wheat plants under salt stress. Salt stress was given after 3 weeks of seed germination. **(A)** Represents reduced glutathione activity; **(B)** represents ascorbate activity; **(C)** represents peroxidase activity; **(D)** represents catalase activity; Ctrl, control plants; Trt, BTK-1 inoculated plants; Means followed by different letters are significantly different at *P* ≤ 0.05. Each bar shows ± SE of means.

Under normal growth conditions (0 mM NaCl stress), BTK-1 association with the wheat plants exhibited no effect on the activities of reduced glutathione (GSH), ascorbate, and peroxidase (Figure 8). However, under NaCl stress wheat plants inoculated with BTK-1 had significantly (*P* = 0.05) higher reduced glutathione (GSH), while the activities of peroxidase, ascorbate, and catalase were significantly (*P* = 0.05) reduced compared to the BTK-1 non-inoculated plants (Figure 8).

### Effect of BTK-1 on Wheat Endogenous Indole Acetic Acid, Gibberellins, and Abscisic Acid Under Salt Stress

**FIGURE 9:**
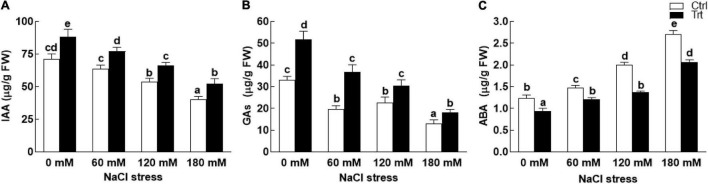
Effect of endophytic fungal isolate BTK-1 on phytohormones of wheat plants under salt stress. Salt stress was given after 3 weeks of seed germination. **(A)** Represents IAA content; **(B)** represents GAs content; **(C)** represents ABA content; IAA, indole acetic acid; GAs, gibberellins; ABA, abscisic acid; Ctrl, control plants; Trt, BTK-1 inoculated plants; Means followed by different letters are significantly different at *P* ≤ 0.05. Each bar shows ± SE of means.

The results revealed that BTK-1 inoculated wheat plants and non-inoculated plants significantly (*P* = 0.05) differed in phytohormones (IAA, GAs, and ABA) contents under normal (0 mM NaCl stress) as well as salt stress (60, 120, and 180 mM) conditions. BTK-1 associated wheat plants exposed to various salt concentrations (60, 120, and 180 mM) showed significantly (*P* = 0.05) high contents of IAA compared to the non-inoculated plants (Figure 9A). Similarly, upon exposure to NaCl stress (60, 120, and 180 mM), a significant (*P* = 0.05) increase in GA contents were found in BTK-1 inoculated wheat plants as compared to non-inoculated plants (Figure 9B). An opposite trend was noticed for ABA contents in wheat plants exposed to salt stress (Figure 9C). The ABA contents were significantly (0.05) low in BTK-1 associated wheat plants compared to the non-inoculated plants under normal (0 mM NaCl stress) as well as salt stress conditions (Figure 9C).

### Effect of BTK-1 on Wheat Mineral Contents Under Salt Stress

**FIGURE 10:**
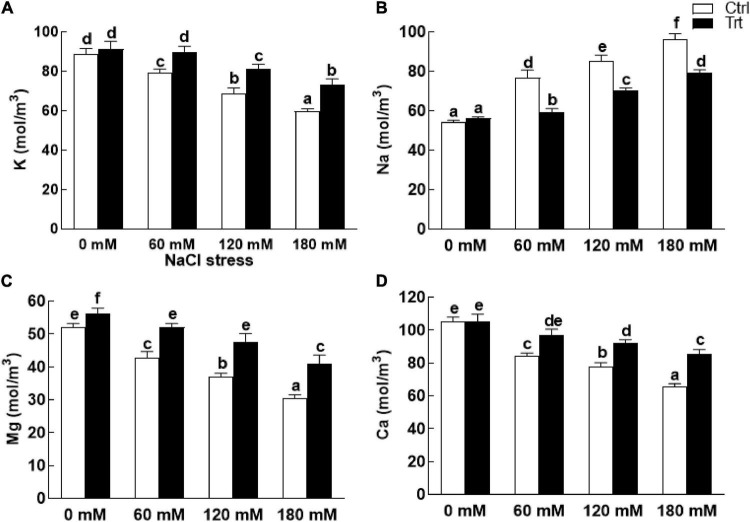
Nutrient quantification of endophytic fungal isolate BTK-1 inoculated and non-inoculated wheat plants under salt stress. Salt stress was given after 3 weeks of seed germination. **(A)** Represents K^+^ concentration; **(B)** represents Na^+^ concentration; **(C)** represents Mg^+^ concentration; **(D)** represents Ca^+^ concentration; K^+^, potassium; Na^+^, sodium; Mg^+^, magnesium; Ca^+^, calcium; Ctrl, control plants; Trt, BTK-1 inoculated plants; Means followed by different letters are significantly different at *P* ≤ 0.05. Each bar shows ± SE of means.

The data recorded for nutrients analysis of wheat plants (with or without fungal inoculation) showed higher levels of K^+^ ions under normal condition (0 mM NaCl stress). However, after exposure to salinity, the levels of K^+^ in BTK-1 non-inoculated wheat plants was significantly lower compared to the BTK-1 inoculated wheat plants (Figure 10A). On the contrary, significantly (*P* = 0.05) high Na^+^ ions were observed in BTK-1 non-inoculated wheat plants compared to the BTK-1 inoculated plants exposed to various concentrations (60, 120, and 180 mM) of NaCl (Figure 10B). Moreover, salt treatments (60, 120, and 180 mM) showed significant (*P* = 0.05) declination in Mg^2+^ and Ca^2+^ ions in BTK-1 non-inoculated wheat plants compared to the BTK-1 inoculated plants (Figures 10C,D).

## Discussion

In connection with the prior investigation, the current research was designed to isolate and screen endophytic fungi for polyphasic potential in order to boost wheat growth. The fungal isolates were screened for hormonal analysis and plant growth properties in order to discover potent isolate(s) (Gul Jan et al., 2019; Ismail et al., 2021). In screening bioassays, maize (IAA mutant and wild maize) seedlings were grown in hydroponic medium containing culture filtrates of isolated endophytic fungus under sterilized conditions. After a week, the best maize mutant growth promoting and IAA producing endophytic fungal isolate BTK-1 was selected and utilized in further experiments. The IAA producing endophytes with an active role in plant growth has been demonstrated by various scientists in recent times (Muhammad et al., 2018, 2019; Mehmood et al., 2019a). In fact, some of the discovered endophytes have shown the ability to secrete higher amounts of IAA, which is one of the important factors that is required by the plants to grow normally under stress conditions (Muhammad et al., 2018, 2019; Mehmood et al., 2019a). The presence of IAA in the CF of isolated BTK-1 suggests that the interaction of this endophytic fungus with wheat plants has stimulated the host growth through IAA secretion under salt stress. Several fungal endophytes, like *Aspergillus flavus*, *Paecilomyces formosus*, *Aspergillus niger*, *Penicillium funiculosum*, *Penicillium corylophilum*, and *Fusarium oxysporum* have been reported for their plant growth promotion and IAA production (Deng and Cao, 2017). Muhammad et al. (2018) reported that *Penicillium roqueforti* have the capacity to produce IAA and help the host plant species under stressful conditions.

Plants, although they are generally regarded as self-sufficient entities, are in reality associated with diverse microbial communities that confer multiple benefits under stress (Ismail et al., 2021). More recently, the role of symbiotic endophytes has been acknowledged by scientists for their role in plant resistance against stress conditions (Hamayun et al., 2017; Bibi et al., 2018). Similarly, reports are available on the role of plant growth promoting endophytes (fungi and bacteria) that increase host plants fitness by mitigating the impacts of abiotic stresses (Hamayun et al., 2017; Bibi et al., 2018). Numerous reports on endophytes suggested that fungal interaction can enhance plants growth under stress situations (Ismail et al., 2021; Raid et al., 2021). In addition to phytoharmones, biofertilization ability of endophytic fungi ensure availability of essential nutrients to host plants (Raid et al., 2021). Colonization potential of endophytes and mechanisms such as phosphate solubilization and siderophores production are very important for the host plant growth under stress (Muhammad et al., 2018, 2019). The current study also rectifies the plant growth promoting properties of the endophytic fungal isolate BTK-1. The inoculation of wheat plants with BTK-1 considerably enhanced wheat growth and helped in relieving induced salt stress (Muhammad et al., 2018; Mehmood et al., 2019a). The occurrence of inoculated endophytic fungal isolate BTK-1 inside cortical region of wheat plant roots and their successful re-isolation further strengthens the active role of BTK-1. Likewise, the antioxidant scavengers can augment membrane firmness and can protect plant species against ROS attack (Ismail et al., 2021). The ROS scavenging activity can be assessed through the production of MDA, which is the lipid breakdown product under stress situations (Ismail et al., 2021). The MDA content reflects the ROS production in plant tissues during stress and is responsible for the instability of cellular membrane. From the results, we observed low levels of MDA content in fungus inoculated wheat plants that might have boosted the membrane thermo stability under induced salinity.

Osmo-protectants (proline) aggregation offer avoidance of osmotic imbalances in plants under stress circumstances (Ismail et al., 2020c,^2021^). Correspondingly, nitrogen uptake by plants regulates the uptake of sodium ions that can lead to maintenance of chlorophyll and ionic stability under saline conditions (Gupta and Huang, 2014). In the current study, substantial accumulation of proline was observed in BTK-1 treated wheat plants grown under induced salinity, which signifies a decline in ionic influx and protects host plants from ionic imbalance (Gupta and Huang, 2014). Sodium and chloride ion toxicity can generate ROS, which can cause destruction in cellular functioning (Gupta and Huang, 2014). On the contrary, aggregation of antioxidants inside plant species can extend greater resistance to oxidative damage (Gharsallah et al., 2016). In the present work, we observed greater activities of antioxidant enzymes (CAT, APX, SOD, and GSH) in BTK-1 inoculated wheat plants, suggesting high oxidative stability under salt stress. Several studies reported the increased antioxidant potential of plant species associated with fungal endophytes under stress conditions (Jan et al., 2019; Muhammad et al., 2019; Ismail et al., 2021). In addition, plant hormones can secure plants and intercept stress injury through various mechanisms (Ismail et al., 2021). It is well-known that ABA levels under stress conditions increase in plants. However, our result showed low levels of ABA in BTK-1 inoculated wheat plants exposed to salt stress. Similarly, Ismail et al. (2020b) also reported low content of ABA in soybean plants associated with endophytic fungi under heat stress. However, the influence of fungi may vary among the species and its interaction with plant species as well (Ahmad et al., 2010; Ismail et al., 2018, 2019, 2020a,b,c, 2021). IAA is also extensively recognized due to its active role in plant growth and developmental processes (Kumar et al., 2017). In the current study, we observed significantly high levels of IAA in endophyte treated wheat plants that might be responsible for normal plant growth under salt stress.

## Conclusion

BTK-1 inoculation and its symbiotic-interaction with wheat plants have promoted the growth and strengthened the host plants against the adverse effects of salinity. BTK-1 as an endophyte has secreted phytoharmones, including IAA and GA, inside the host plant that improved the productivity and quality of economically important wheat crop under stress conditions.

## Data Availability Statement

The datasets presented in this article are not readily available because all the data included in the manuscript. Requests to access the datasets should be directed to AI, amjadiqbal@awkum.edu.pk.

## Author Contributions

MK, GJ, and FJ designed and performed all the experiments. AI and AH analyzed the data and wrote the manuscript. AI, AH, and MH designed the experiments. AI, NA, and MH edited the manuscript. NA, GJ, MH, and I-JL supervised the project. MH and I-JL arranged the resources for the work. I-JL financed the project. All the authors contributed to the article and approved the submitted version.

## Conflict of Interest

The authors declare that the research was conducted in the absence of any commercial or financial relationships that could be construed as a potential conflict of interest.

## Publisher’s Note

All claims expressed in this article are solely those of the authors and do not necessarily represent those of their affiliated organizations, or those of the publisher, the editors and the reviewers. Any product that may be evaluated in this article, or claim that may be made by its manufacturer, is not guaranteed or endorsed by the publisher.

## Footnotes

1
http://www.ncbi.nlm.nih.gov/
